# Four New Species of the Genus *Eoneureclipsis* (Trichoptera: Psychomyiidae) from China Inferred from Morphology and DNA Barcodes

**DOI:** 10.3390/insects14020158

**Published:** 2023-02-04

**Authors:** Lang Peng, Haoming Zang, Changhai Sun, Le Wang, Beixin Wang

**Affiliations:** 1 Lab of Insect Taxonomy & Aquatic Insects, College of Plant Protection, Nanjing Agricultural University, Nanjing 210095, China; 2 Nanjing Institute of Environmental Sciences, Ministry of Ecology and Environment of China, Nanjing 210042, China

**Keywords:** Oriental Region, caddisfly, psychomyiids, morphology, *COI*

## Abstract

**Simple Summary:**

The family Psychomyiidae Walker, 1852 currently includes 8 genera and more than 600 extant species, and is represented by three subfamilies, Psychomyiinae, Tinodinae, and Eoneureclipsinae. Subfamily Eoneureclipsinae was established by Mey in 2013, with the genus *Eoneureclipsis* Kimmins, 1955, based on morphological features, which were significant enough to separate it from the other genera of family Psychomyiidae. The new species in this study provide additional distribution data to the genus.

**Abstract:**

Four new species of the genus *Eoneureclipsis* Kimmins, 1955 from China are described, illustrated, and diagnosed based on male genitalia: *Eoneureclipsis jianfenglingensis* sp. nov. from Hainan, *E. foraminulatus* sp. nov. from Guangxi, *E. spinosus* sp. nov. from Guangxi and Guangdong, and *E. gei* sp. nov. from Fujian. A dichotomous key to Chinese adult males of *Eoneureclipsis* is provided. A distribution map for all *Eoneureclipsis* species is also presented. The DNA barcodes (partial mt*COI* sequences) of *E. jianfenglingensis* sp. nov., *E. gei* sp. nov., and *E. hainanensis* Mey, 2013 have been generated and compared with all existing sequences of *Eoneureclipsis* species.

## 1. Introduction

**Figure 1:**
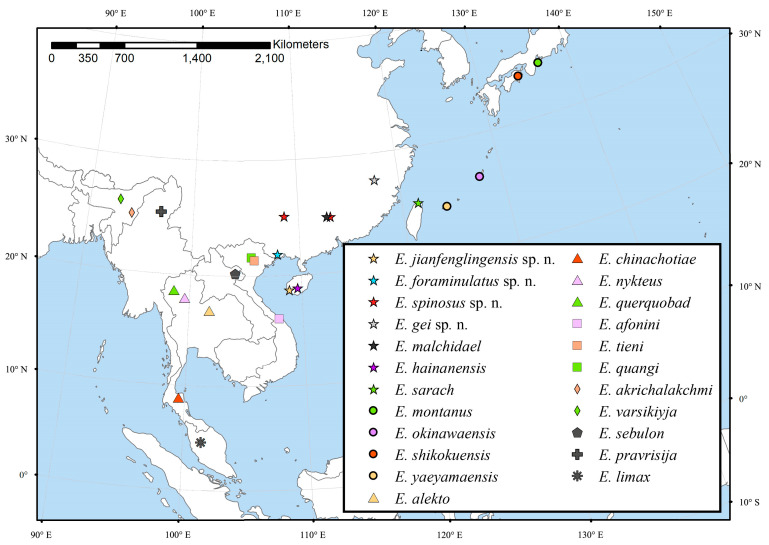
Distribution map of *Eoneureclipsis* species.

The genus *Eoneureclipsis* Kimmins, 1955 [1] is a small genus in the family Psychomyiidae Walker, 1852 [2,3], containing 19 [4,5,6] extant species with two species distributed in the Eastern Palearctic Region, Japan [7] and 17 in the Oriental Region, of which, four species are distributed in Thailand [5,8,9,10,11], three in Vietnam [12,13,14], three in China [6,15,16], two species each in India [17] and Japan [7], and one species each in Laos [18], Myanmar [17], and Malaysia [1]. Their distributions are shown in Figure 1.

Recognition of the *Eoneureclipsis* larvae occurred much later than that for adults. Larvae suspected to be of the genus *Eoneureclipsis* were first recorded in 2005 by Thamsenanupap et al. [19]; they found that the larvae of two species from northern Thailand had a larger body size than the larvae of other genera among the psychomyiids and treated them as *Eoneureclipsis querquobad* Malicky & Chantaramongkol, 1989 and *Eoneureclipsis alekto* Malicky & Chantaramongkol, 1997 according to their distributions. Torii and Nakamura [20] in 2016 associated a putative *Eoneureclipsis* larva with its adult by using mt*COI* sequence data and described it in detail.

Up to now, three *Eoneureclipsis* species have been reported in China. The first one was recognized by Mey [15] from Hainan; in his research, he established a new subfamily Eoneureclipsinae to accommodate all *Eoneureclipsis* species. The second and third species were described as from Taiwan [16] and Guangdong [6] by Malicky (Table 1).

In this study, we describe four new species from Oriental China based on male adults, bringing the world’s *Eoneureclipsis* fauna to 23 (Table 1).

## 2. Materials and Methods

### 2.1. Sample Collection

**Figure 2:**
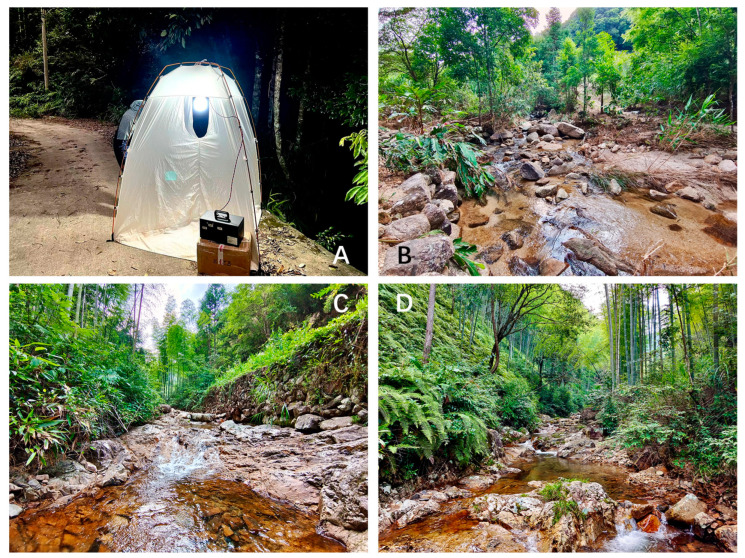
Photograph of a mobile light trap (**A**) and photographs of ecosystems (**B**–**D**): (**A**) light trap with mobile power and a high-pressure mercury lamp, in Hainan; (**B**) a tributary in Jianfengling National Forest Park, Hainan Province; (**C**,**D**) the upper tributary of Dazhulan, Wuyishan National Park, Fujian (photographs by Haoming Zang and Lang Peng).

A total of 8 adult specimens of the new species were collected in 2004, 2019, and 2022 with light traps (Table 2). After collection, they were preserved in 95% ethanol in the field. A photograph of a mobile light trap and photographs of the ecosystems (Figure 2) were taken with an Olympus TOUGH TG-5 digital camera.

### 2.2. Morphological Study

The methods used for the preparation of specimens followed Xu et al. [21]. Male abdomens used for illustrations were cleared with 10% NaOH solution and heated to 80 °C for 20 min to remove all the non-chitinous tissues. Then the cleaned genitalia were rinsed in distilled water and mounted on a depression slide with lactic acid for examination. Genitalic structures of males were traced with the pencil using a Nikon Eclipse 80i microscope equipped with a camera lucida. Pencil drawings were scanned with an Epson Perfection (V30 SE) scanner, then placed as templates in Adobe Photoshop (Version: CC 2018 19.0) and inked digitally with a Wacom CTL-671 tablet to produce the final illustrations. Then each abdomen was stored in a microvial together with the remainder of the specimen in 95% ethanol.

All specimens have been deposited in the Insect Collection, Nanjing Agricultural University, Nanjing, Jiangsu Province, China (NJAU).

The terminology for male genitalia mainly follows that of Mey [15], and the term “ventral plate” is used to refer to the expanded ventral lobe in the apical portion of the phallic apparatus underneath the aedeagus. The terminology for wing venation follows that of Torii and Nishimoto [7].

### 2.3. Molecular Analysis

The left hind legs of three individuals (males) were taken from the body for DNA extractions. DNA extraction and PCR amplification followed the procedures of Zang [22]. The primers [23] (LCO1490/HCO2198) used to amplify the 658 bp fragment of the mitochondrial (mt) cytochrome c oxidase I unit (*COI*) are listed in Table 3.

Fragment sequencing and analysis followed the procedures of Ge [24]. Raw sequences were assembled and edited in Sequencher 4.5 (Gene Codes Corporation, Ann Arbor, MI, USA). A neighbor-joining (NJ) tree of 32 species within the family Psychomyiidae was constructed using MegaX v10.2.6 [25], with the following parameters: Kimura 2-parameter substitution model (K2P) [26], pairwise gap deletion, and others as defaults. The same software was used to calculate the K2P corrected *p*-distance of the 658 bp mt*COI* fragment among all *Eoneureclipsis* species sequences available (Table S1). *COI* sequences of three *Eoneureclipsis* species were uploaded to GenBank. Accession numbers of the other analyzed *Eoneureclipsis* species are shown in Table S1.

## 3. Results

### 3.1. Taxonomy

Family Psychomyiidae Walker, 1852

Genus *Eoneureclipsis* Kimmins, 1955

#### 3.1.1. *Eoneureclipsis jianfenglingensis* Peng, Zang & Sun, sp. nov. (Figure 3A–E and Figure 7A)

Description: Length of each forewing 6.9–7.0 mm (n = 2). Specimens (in alcohol) brown, with compound eyes black, antennae uniformly yellowish brown, palpi pale brown, legs and spurs yellowish brown, and wings dark brown. Both pairs of wings elliptical, each with rounded apex; each hind wing with costal margin, not sinuate and without projection as in other psychomyiid genera (Figure 7A). Venation typical for the genus. Forewings each with Forks I, II, III, IV, and V present, discoidal cell short, median and thyridial cells relatively elongate; irregular hyaline area across *r-m*, *m*, *m-cu*, and middle of M; two nygmas located at bases of R_4_ cell (Fork II) and thyridial cell (TC). Hind wings each with Forks I, II, III, and V present; one nygma present at base of R_4_ cell (Fork II).

**Figure 3:**
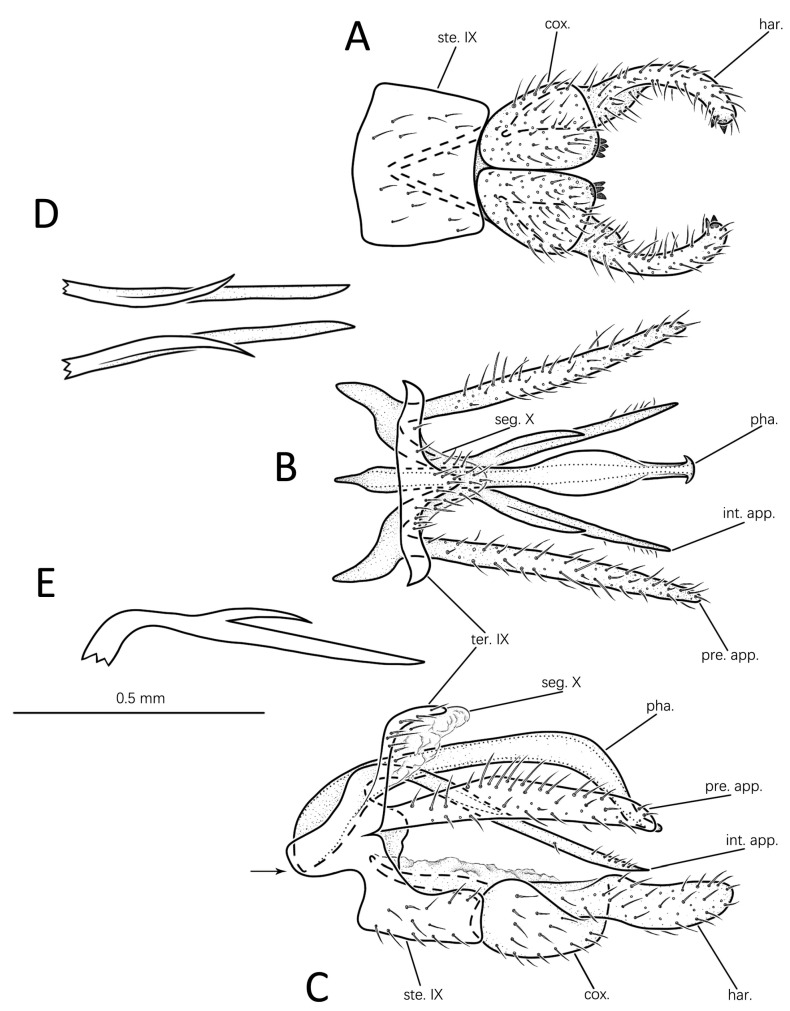
Male genitalia of *Eoneureclipsis jianfenglingensis* sp. nov.: (**A**) ventral; (**B**) dorsal; (**C**) left lateral; (**D**) intermediate appendages of paratype, dorsal; (**E**) one intermediate appendage of paratype, left lateral. Abbreviations: ste. IX = sternum IX; ter. IX = tergum IX; seg. X = segment X; pre. app. = preanal appendages (paired); int. app. = intermediate appendages (paired); cox. = coxopodite (paired); har. = harpago (paired); pha. = phallic apparatus. Scale bar refers to **A**–**E**.

Male genitalia: Tergite IX hairy, slender, nearly vertical, bent posterodorsad from segment’s mid-height in lateral view (Figure 3C); posterior margin produced into mesal triangular protrusion in dorsal view (Figure 3B). Sternite IX well developed, trapezoid in ventral view (Figure 3A); nearly rectangular with anterodorsal angle produced into projections in lateral view (Figure 3C). Segment X membranous, triangular, extending beyond apex of tergite IX in dorsal view (Figure 3B); irregular in lateral view (Figure 3C). Preanal appendages long, setose, straight, stick-shaped in dorsal view (Figure 3B); boomerang-shaped in lateral view (Figure 3C), each widest at middle, narrower at base and apex. Intermediate appendages originating laterally from anterolateral bases of sternite IX, inclining posteroventrad in lateral view (Figure 3C); straight, slender in dorsal view (Figure 3B), slightly shorter than preanal appendages, each with elongate process near base, straight in dorsal and lateral views (Figure 3B,C). Inferior appendages stout, setose, each with distal end of harpago extending beyond phallic apparatus in lateral view (Figure 3C); coxopodites closely appressed in ventral view (Figure 3A), each rectangular with anterodorsal angle produced internally into elongate apodeme (basal plate); and each with several stout spines at apicomesal corner (Figure 3C). Harpagones elongate, each with basal 1/3 fused with distal half of coxopodites in lateral view (Figure 3C); in ventral view (Figure 3A) with apex bent mesad, each with two spines apically. Phallic apparatus long, arched, with base slightly swollen, and distal 1/4 posteroventrad in lateral view (Figure 3C); basal half tubular, distal half swollen and suddenly narrowed, with apex having back hooks laterally in dorsal view (Figure 3B).

Diagnosis: The species is similar to *Eoneureclipsis yaeyamaensis* Torii & Nishimoto, 2011 from Japan in the intermediate appendages in which each has one short branch. However, the new species can be easily distinguished from the latter by the following: (1) the preanal appendages are elongate-triangular in lateral view, rather than clavate and curved at the base as in *E. yaeyamaensis*; (2) the intermediate appendages are branched near the base, rather than branched subapically as in *E. yaeyamaensis*; and (3) the coxopodites are rectangular in ventral view, rather than elliptical as in *E. yaeyamaensis*.

Holotype: Male, P.R. China, Hainan Province: Ledong Li Autonomous County, Jianfeng Town, Jianfengling National Forest Park, Jianfeng Parking Lot, 18.7107° N, 108.8758° E, alt. 706 m, 16 Apr 2019, light trap, leg. H. Song (NJAU).

Paratype: P.R. China, Hainan Province: one male, Ledong Li Autonomous County, Jianfeng Town, Jianfengling National Forest Park, bridge over a tributary, 0.5 km east of Jianfeng Parking Lot, 18.7087° N, 108.8844° E, alt. 920 m, 27 Jul 2022, light trap, leg. L. Peng & H. Zang (NJAU).

Distribution: China (Hainan).

Etymology: The name is derived from that of the locality type: *Jianfengling* mountain, Hainan Province.

Remarks: The elongate sub-basal processes of the intermediate appendages in the paratype are curved outward in dorsal view (Figure 3D), each elevated at an angle above the trunk of its intermediate appendages in lateral view (Figure 3E).

#### 3.1.2. *Eoneureclipsis foraminulatus* Peng, Zang & Sun, sp. nov. (Figure 4A–C and Figure 7B)

Description: Length of each forewing 8.1–8.2 mm (n = 1). Specimen in alcohol with compound eyes black; antennae, palpi, legs, spurs, and wings pale yellow; other parts of body yellow. Venation typical for genus, similar to that of *E. jianfenglingensis* sp. nov. Wings uniformly brown, without transparent patches (Figure 7B).

**Figure 4:**
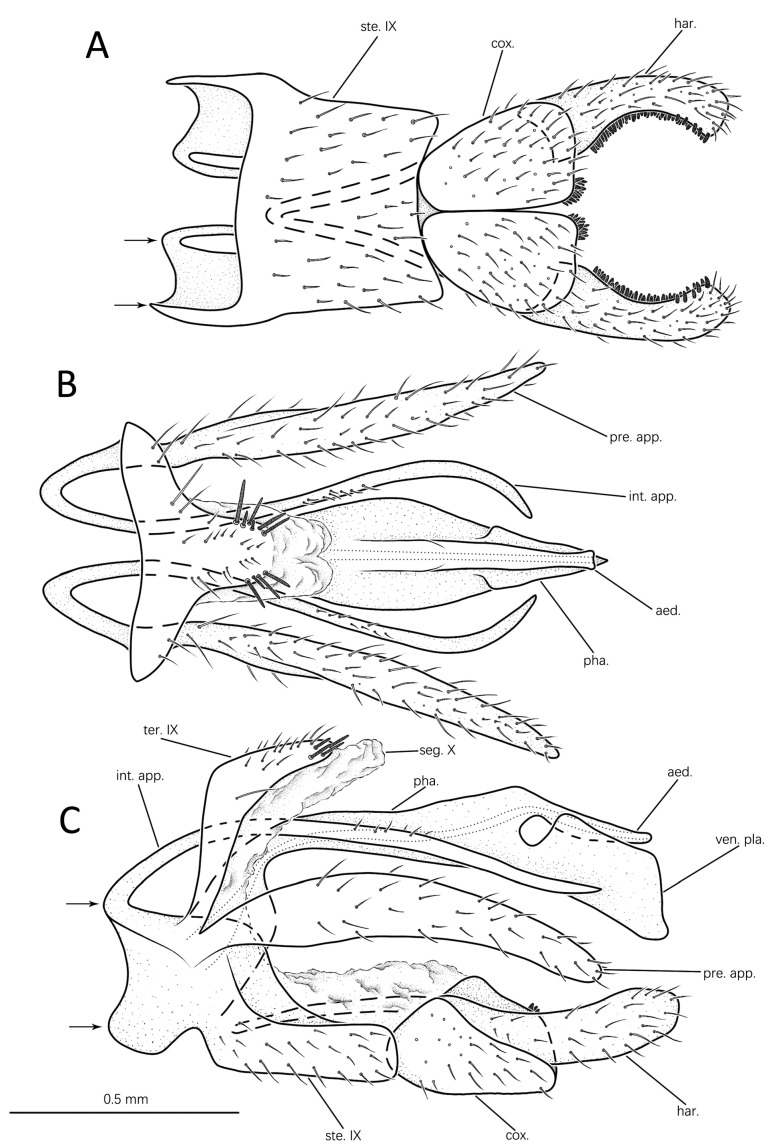
Male genitalia of *Eoneureclipsis foraminulatus* sp. nov.: (**A**) ventral; (**B**) dorsal; (**C**) left lateral. Abbreviations: ste. IX = sternum IX; ter. IX = tergum IX; seg. X = segment X; pre. app. = preanal appendages (paired); int. app. = intermediate appendages (paired); cox. = coxopodite (paired); har. = harpago (paired); pha. = phallic apparatus; aed. = aedeagus; ven. pla. = ventral plate. Scale bar refers to **A**–**C**.

Male genitalia: Tergite IX nearly vertical, boomerang-shaped in lateral view (Figure 4C); posterior margin strongly expanded into blunt apicodorsal lobe, with several long ossifying spines on each side in dorsal view (Figure 4B). Sternite IX well developed, trapezoidal in ventral view (Figure 4A) with anterolateral angles produced; nearly rectangular, with anterodorsal angles markedly enlarged in lateral view (Figure 4C). Segment X membranous, extending beyond apex of tergite IX, with apex shallowly incised in dorsal view (Figure 4B); bordering tergite IX and extending beyond it in lateral view (Figure 4C). Preanal appendages lanceolate, setose in dorsal view (Figure 4B); each curved and clavate with narrow base in lateral view (Figure 4C). Intermediate appendages originating from anterolateral bases of sternite IX, about same length as preanal appendages, tilted downward in lateral view (Figure 4C); clamp-shaped (basally divergent and apically curved mesad) in dorsal view (Figure 4B). Inferior appendages sturdy, pilose in lateral view (Figure 4C). Coxopodites each subtriangular in ventral view (Figure 4A), with cluster of apicomesal teeth; subtriangular in lateral view (Figure 4C), each with anterodorsal angle produced into long apodeme. Harpagones elongate, each with apex rounded, bent slightly upward in lateral view (Figure 4C); inner surface with row of dense short spines and bent apicomesad in ventral view (Figure 4A). Phallic apparatus in lateral view long, arched, basal and apical portions wider than middle, apical portion divided into dorsal tubular aedeagus and ventral trapezoidal plate with round hole between them basally (Figure 4C); spindle-shaped in dorsal view, aedeagus slightly constricted subapically, ventral plate triangular (Figure 4B); and V-shaped in caudal view.

Diagnosis: This species is similar to *Eoneureclipsis afonini* Arefina-Armitage & Armitage, 2015 from Vietnam. However, the new species can be easily distinguished from the latter by the following: (1) the apex of the apicodorsal lobe of tergite IX is rounded rather than incised as in *E. afonini*; (2) the paired intermediate appendages are curved in the shape of a clamp in dorsal view but are straight in *E. afonini*; and (3) the phallic apparatus is spindle-shaped in dorsal view rather than tapering from base to apex in *E. afonini*.

Holotype: Male, P.R. China, Guangxi Province: Fangchenggang City, Shangsi County, Shiwandashan National Forest Park, a fourth tributary of Shitou He, 3.8 km southwest of main entrance to Park, 21.8914° N, 107.9047° E, alt. 420 m, 06 Jun 2004, light trap, leg. J.C. Morse & C. Sun (NJAU).

Distribution: China (Guangxi).

Etymology: The Latin adjective *foraminulatus* means foraminate, referring to the small circular aperture formed between the aedeagus and ventral plate at their bases in lateral view.

#### 3.1.3. *Eoneureclipsis spinosus* Peng, Zang & Sun, sp. nov. (Figure 5A–F and Figure 7C)

Description: Length of each forewing 7.4–9.4 mm (n = 3). Specimens in alcohol with compound eyes black; antennae, palpi, and wings pale yellow; legs, spurs, and abdomen yellowish brown; thorax dark brown. Venation typical for genus, similar to that of *E. jianfenglingensis* sp. nov. Wing surface uniformly brown, without transparent patches (Figure 7C).

**Figure 5:**
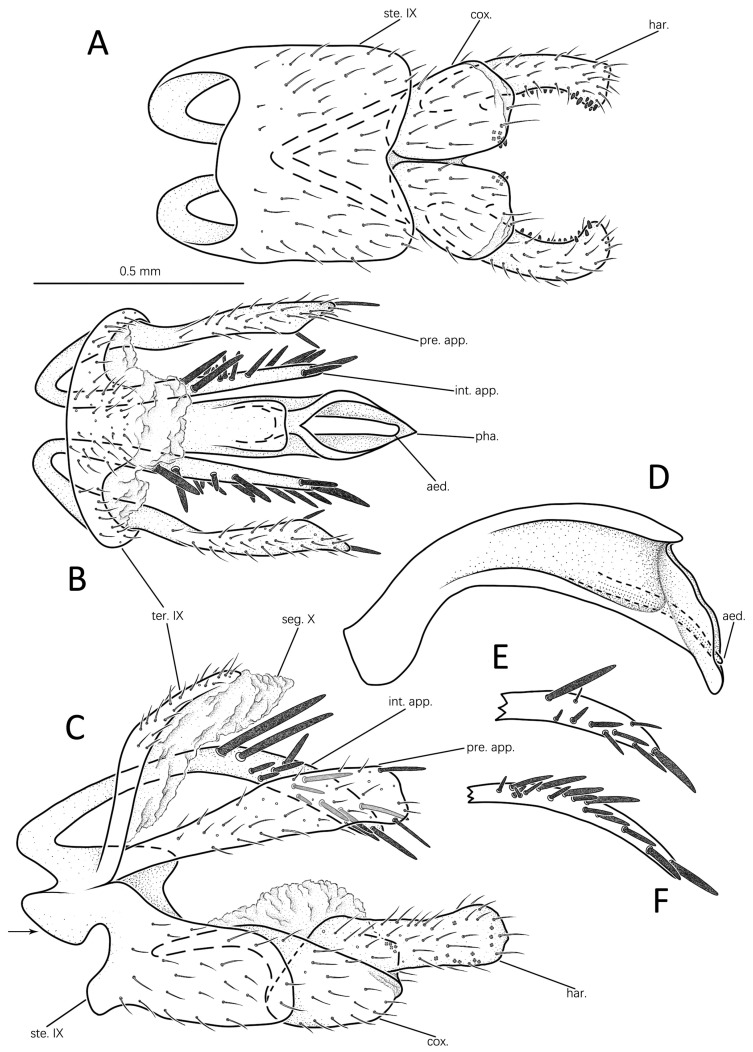
Male genitalia of *Eoneureclipsis spinosus* sp. nov.: (**A**) ventral; (**B**) dorsal; (**C**) left lateral; (**D**) phallic apparatus, left lateral; (**E**) intermediate appendages of paratype (Guangxi), left lateral; (**F**) intermediate appendages of paratype (Guangdong), left lateral. Abbreviations: ste. IX = sternum IX; ter. IX = tergum IX; seg. X = segment X; pre. app. = preanal appendages (paired); int. app. = intermediate appendages (paired); cox. = coxopodite (paired); har. = harpago (paired); pha. = phallic apparatus; aed. = aedeagus. Scale bar refers to **A**–**F**.

Male genitalia: Tergite IX slender, pilous, nearly vertical in lateral view (Figure 5C); posterior margin produced into blunt apicodorsal lobe in dorsal view (Figure 5B). Sternite IX well developed, nearly square in ventral view (Figure 5A); in lateral view (Figure 5C), subrectangular, with each anterodorsal angle produced into irregular process with which each intermediate appendage is articulated. Segment X membranous, extends beyond apex of tergite IX in dorsal view (Figure 5B); irregular in lateral view (Figure 5C). Preanal appendages clavate, long, setose, slightly curved near base in dorsal view (Figure 3B); each with base narrow and apex truncate and with strong spines apicodorally and apicoventrally in lateral view (Figure 5C). Intermediate appendages straight, clavate in dorsal view (Figure 5B); arched in lateral view (Figure 5C), each with distal half having strong spines of varied sizes and number (13–14 in holotype; 10–15 in paratypes) randomly arranged on dorsal and lateral surfaces (Figure 5B). Inferior appendage stout, hairy, extending beyond phallic apparatus in lateral view (Figure 5C). Coxopodites with basal inner halves almost touching in ventral view (Figure 5A), with several small teeth apicomesally; spoon-shaped in lateral view, with anterodorsal angles each produced into apodeme extending deeply into sternite IX (Figure 5C). Harpagones elongate, each with basal half fused with its coxopodite in lateral view and with middle portion slightly constricted (Figure 5C); bent apicomesally in ventral view (Figure 5A); each with inner margin having a row of spines (Figure 5A,C). Phallic apparatus well developed, slightly curved, gradually enlarged from base to apex, apex sinuate in lateral view (Figure 5C), in dorsal view slightly constricted at middle, apex with sloped opening exposing aedeagus (Figure 5B).

Diagnosis: The species is similar to *Eoneureclipsis okinawaensis* Torii & Nishimoto, 2011 from Japan in the shape of the phallic apparatus when viewed laterally but differs from the latter in the following: (1) the preanal appendages are straight, each with its basal half slender in lateral view (clavate and curved in the middle, almost of equal width in *E. okinawaensis*); (2) the distal half of each preanal appendage has strong spines (without spines in *E. okinawaensis*); and (3) the intermediate appendages are unbranched (branched subapically in *E. okinawaensis*).

Holotype: Male, P.R. China, Guangxi Province: Hechi City, Huanjiang County, Jiuwandashan Provincial Nature Preserve, an unnamed tributary of Yangmeiao Xi, 100 m upstream of County Road 5309 marker 124.9 km, 25.1976° N, 108.6494° E, alt. 1155 m, 15 June 2004, light trap, leg. J.C. Morse & C.J. Geraci (NJAU).

Paratypes: P.R. China, Guangxi Province: one male, same data as holotype. P.R. China, Guangdong Province: one male, Shaoguan City, Ruyuan County, Nanling National Nature Preserve, an unnamed tributary of Laopengkeng, Route X327, marker 17.45 km, 24.9128° N, 113.0342° E, alt. 935 m, 21–22 May 2004, light trap, leg. J.C. Morse & C. Sun (NJAU).

Distribution: China (Guangxi, Guangdong).

Etymology: The Latin adjective *spinosus* means spiny, referring to the intermediate appendages with many spines.

Remarks: The spine number of the intermediate appendages varies among individuals. In the paratype from Guangxi, the number is 10 (Figure 5E), but, in the paratype from Guangdong, the number is 15 (Figure 5F).

#### 3.1.4. *Eoneureclipsis gei* Peng, Zang & Sun, sp. nov. (Figure 6A–D and Figure 7D)

Description: Length of each forewing 7.4–7.5 mm (n = 1). Specimen in alcohol with compound eyes black; antennae and palpi pale yellow; legs, spurs, and abdomen yellowish brown; thorax and wings dark brown. Venation typical for genus, similar to that of *E. jianfenglingensis* sp. nov. Forewings with irregular hyaline area across *r-m*, *m*, *m-cu*, and middle of M and small transparent spots irregularly scattered on anterior portions of fore- and hind wings (Figure 7D).

**Figure 6:**
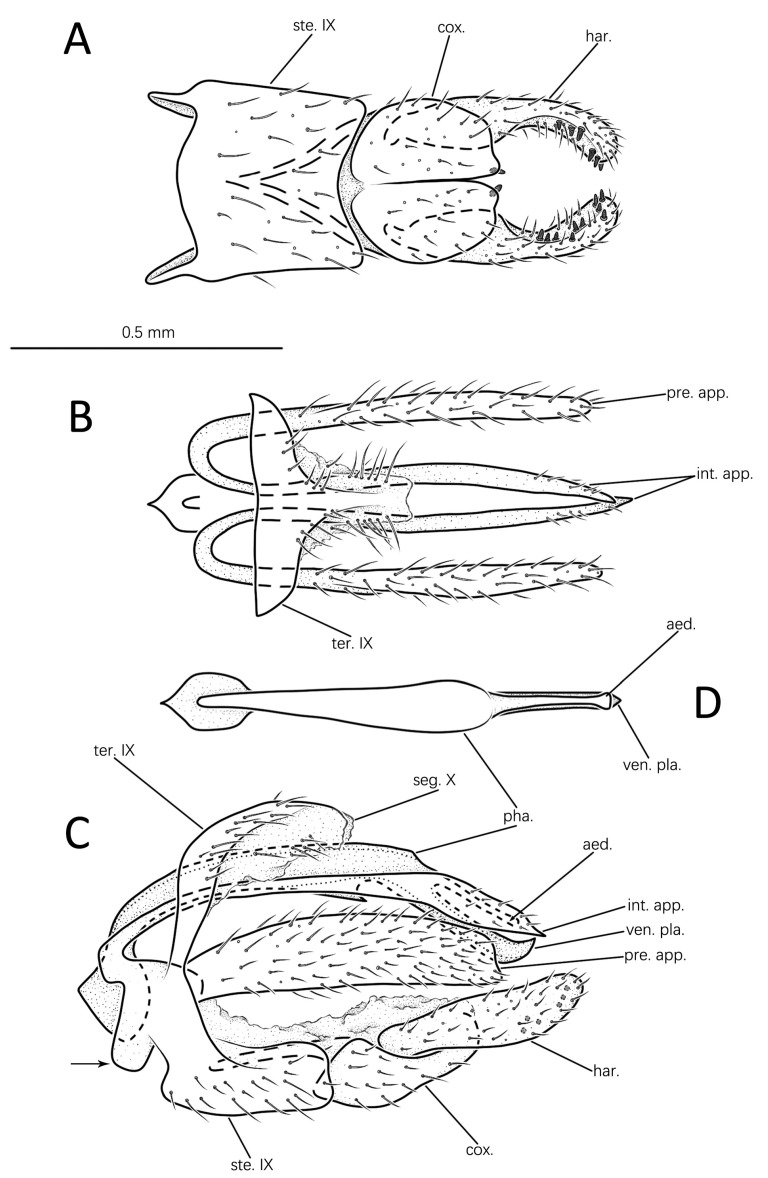
Male genitalia of *Eoneureclipsis gei* sp. nov.: (**A**) ventral; (**B**) dorsal; (**C**) left lateral; (**D**) phallic apparatus, dorsa. Abbreviations: ste. IX = sternum IX; ter. IX = tergum IX; seg. X = segment X; pre. app. = preanal appendages (paired); int. app. = intermediate appendages (paired); cox. = coxopodite (paired); har. = harpago (paired); pha. = phallic apparatus; aed. = aedeagus; ven. pla. = ventral plate. Scale bar refers to **A**–**D**.

**Figure 7:**
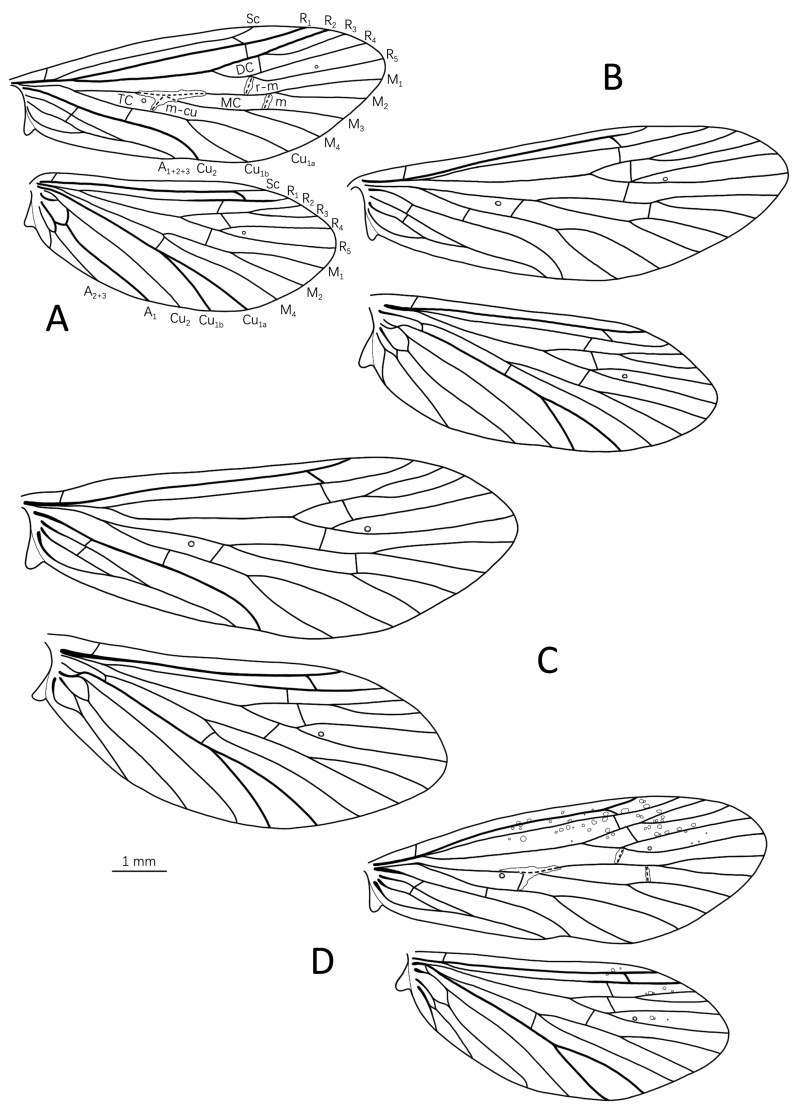
Wing venations of *Eoneureclipsis* species: (**A**) *Eoneureclipsis jianfenglingensis* sp. nov.; (**B**) *E. foraminulatus* sp. nov.; (**C**) *E. spinosus* sp. nov.; (**D**) *E. gei* sp. nov. Abbreviations: Sc = subcosta; R = radius; M = media; Cu = cunitus; A = anal; DC = discoidal cell; MC = medial cell; TC = thyridial cell. Scale bar refers to **A**–**D**.

Male genitalia: Tergite IX slender, setose, nearly vertical in lateral view (Figure 6C); somewhat T-shaped in dorsal view, with produced apicodorsal lobe fused with segment X (Figure 6B). Sternite IX well developed, trapezoid in ventral view (Figure 6A); subrectangular, with each anterodorsal angle produced into irregular projection in lateral view (Figure 6C). Segment X membranous, in dorsal view, with apex slightly incised mesally and setose laterally (Figure 6B); two sides extending downward and enclosing phallic apparatus in lateral view (Figure 6C). Preanal appendages clavate, setose in dorsal view (Figure 6B); elongate-rectangular in lateral view, each with apex truncate (Figure 6C). Intermediate appendages originating from produced projections of sternite IX, in lateral view longer than preanal appendages, each with basal 2/3 narrow and apical 1/3 enlarged, distal end sharp in lateral view (Figure 6C); in dorsal view (Figure 6B) slightly bent mesad, with apices crossed. Inferior appendages stout, hairy in lateral view (Figure 6C). Coxopodites approximately semicircular, their inner margins almost touching along full length in ventral view, each with single spine apicomesally (Figure 6A); subtriangular, long, and upcurved apically in lateral view (Figure 6C). Harpagones elongate-clavate, basal two-fifths fused with coxopodites in lateral view (Figure 6C); somewhat parentheses-shaped in ventral view, with several small spines scattered randomly on inner surfaces in both ventral and lateral views (Figure 6A,C). Phallic apparatus long, slightly curved in lateral view (Figure 6C), lower margin with hook-like incision near 1/3 from apex; in dorsal view (Figure 6D) middle portion swollen; ventral plate protruding upward to acute apex-like scimitar in lateral view (Figure 6C); aedeagus in dorsal view straight and slender, issuing dorsally at 1/3 from apex of phallic apparatus, with its apex slightly swollen, parallel to apicoventral plate in lateral view (Figure 6C); narrower in dorsal view (Figure 6D).

Diagnosis: The species is similar to *Eoneureclipsis varsikiyja* Schmid, 1972 from India in the shape of the intermediate appendages in lateral view but differs from the latter in that (1) the preanal appendages are elongate-rectangular in lateral view (slightly enlarged basally in *E. varsikiyja*); (2) the dorsal margin of the phallic apparatus is slightly arched in lateral view (strongly sinuate in *E. varsikiyja*); and (3) the ventral margin of the phallic apparatus has a semicircular incision in lateral view (nearly straight in *E. varsikiyja*).

Holotype: Male, P.R. China, Fujian Province: Wuyishan City, Xingcun Town, Wuyishan National Park, the upper tributary of Dazhulan 27.6985° N, 117.6521° E, alt. 884 m, 16 July 2022, light trap, leg. L. Peng, H. Zang, X. Ge & C. Sun (NJAU).

Distribution: China (Fujian).

Etymology: The new species is named after Mr. Xinyu Ge in honor of his unprecedented contributions to the study of mitochondrial genomics and phylogenetic research in Trichoptera.

### 3.2. Key to Male Chinese Eoneureclipsis Species

With four new species in this study, a total of seven *Eoneureclipsis* species occur in China. A concise key to these seven species is presented below.

1 Preanal appendages each with apex truncate ([16], Figure 5C) or broad (Figure 6C) in lateral view ……………………………………………………………………………….2
- Preanal appendages acute or narrow in lateral view ……………………………………4
2 Intermediate appendages with stout spines (Figure 5B,C,E) ……*E. spinosus* sp. nov.
- Intermediate appendages without stout spines ([16], Figure 6B,C) …………………..3
3 Intermediate appendages longer than phallic apparatus, slender, each curved upward subapically in lateral view [16] ……………………………………………*E. sarach*
- Intermediate appendages of the same length as phallic apparatus, each broad subapically then abruptly tapered to acute apex in lateral view (Figure 6C) …*E. gei* sp. nov.
4 Phallic apparatus simple, without ventral plate (Figure 3B,C); intermediate appendages each with elongate process (Figure 3B–E) ……………*E. jianfenglingensis* sp. nov.
- Phallic apparatus with ventral plate; intermediate appendages simple ………………5
5 Phallic apparatus with dorsal process basally in lateral view [6].…………*E. malchidael*
- Phallic apparatus not as above ([15], Figure 4C) …………………………………………6
6 Tergite IX posterior margin strongly expanded, with several long ossifying spines on each side in dorsal view (Figure 4B) ……………………………*.E. foraminulatus* sp. nov.
- Tergite IX tapering to end, without spines in dorsal view [15] …………*E. hainanensis*

### 3.3. Molecular Analysis

**Figure 8:**
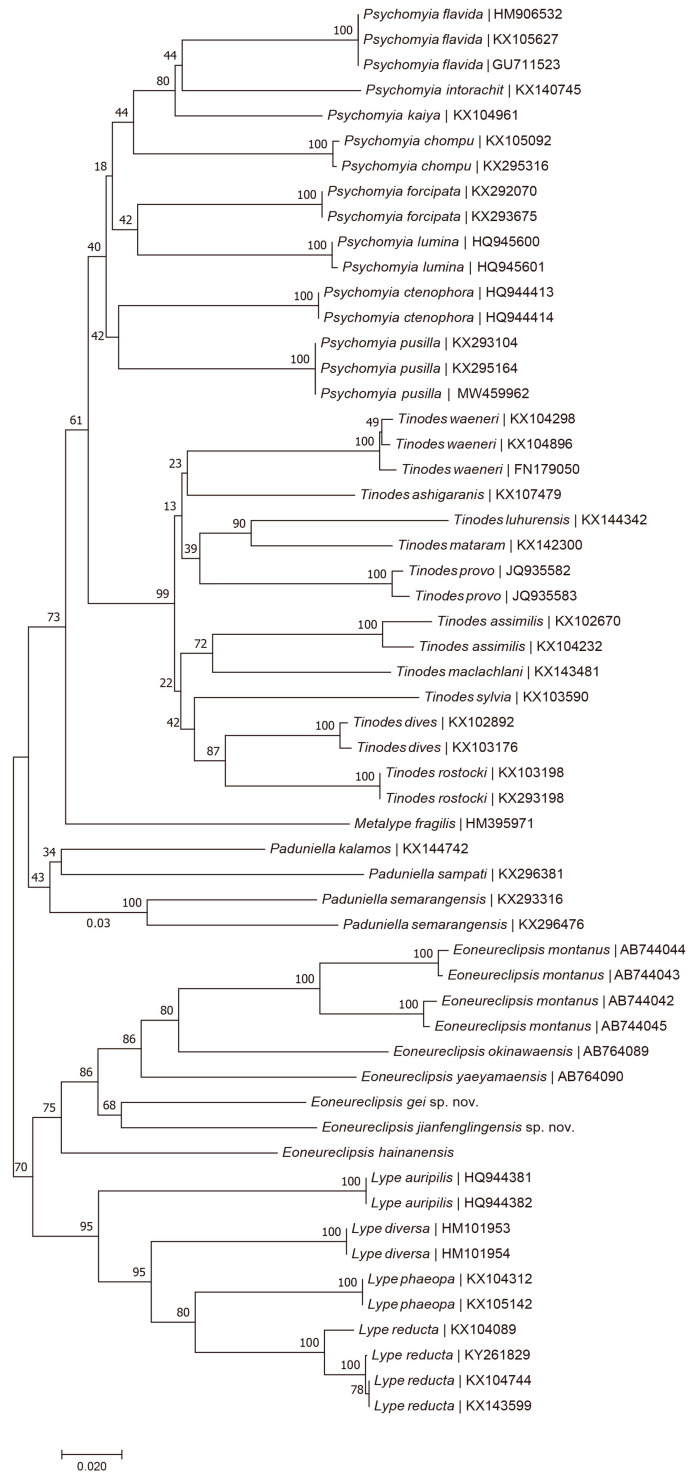
Neighbor-joining cladogram based on the 658 bp long mt*COI* sequence of six *Eoneureclipsis* species. Numbers on branches represent the bootstrap values based on 1000 replicates; scale bar indicates Kimura’s 2-parameter genetic distance of 0.02.

DNA sequences were aligned for 56 extracts of 32 psychomyiid species with a full barcode length of 658 base pairs. For the genus *Eoneureclipsis*, a single clade was well supported in the NJ tree (Figure 8). The interspecific distances (K2P *p* value) between specimens of genus *Eoneureclipsis* and species of other genera were more than 17.60% (Table S1). Based on existing DNA barcodes, the minimum interspecific divergence was 12.60% between *E. okinawaensis* and *E. montanus*, and the maximum interspecific divergence was 20.60% between *E. montanus* and *E. hainanensis*. The mean interspecific divergence was 17.10% in the genus *Eoneureclipsis*. Among them, the interspecific divergence of the *Eoneureclipsis* species distributed in Japan ranged from 14.30% to 16.70%, with a mean divergence of 15.91%; *Eoneureclipsis* species distributed in China ranged from 12.60% to 15.90%, with a mean divergence of 14.33%. The mean intraspecific divergence of the *Eoneureclipsis* species was 5.47% with a minimum of 0.5% and a maximum of 8.20% in *E. montanus*; it seems that *E. montanus* perhaps has diverged into two geographic populations.

## 4. Discussion

The genus *Eoneureclipsis* was established by Kimmins within Polycentropodidae to accommodate new species *Eoneureclipsis limax* Kimmins, 1955 from Borneo [1]. Schmid [17] added three Indian species to *Eoneureclipsis* and transferred the genus to Psychomyiidae based on primitive wing venation, the primitive inferior appendages of the male genitalia, and the female ovipositor and regarded the group as a primitive lineage of the family Psychomyiidae [15]. In 1997, Li and Morse [27] performed a phylogenetic analysis on the relationships of six genera of Psychomyiidae based on adult and larval morphology; they suggested that the family Psychomyiidae consists of two subfamilies, i.e., Psychomyiinae and Tinodinae, and treated the subfamily Paduniellinae as a synonym of Psychomyiinae. However, they failed to include the genus *Eoneureclipsis* in their analysis, and the taxonomic position of the genus *Eoneureclipsis* has remained unsolved [28]. In 2013, based on the large body size and other features, Mey [15] established a new monotypic subfamily Eoneureclipsinae to include the genus *Eoneureclipsis* and its members. Thamsenanupap et al. [19] and Torii and Nakamura [20] proved that the larvae also have larger body sizes than those of other psychomyiid genera, clearly different from Psychomyiinae and Tinodinae [29]. Silva’s analysis on the relationship of Xiphocentronidae and Psychomyiidae indicated that the subfamily Eoneureclipsinae was the first clade of the two families with *Lype* placed as a clade right after *Eoneureclipsis* [30]. Our neighbor-joining cladogram based on the 658 bp long mt*COI* sequence of six *Eoneureclipsis* species is a little bit different from the result of Silva (Figure 8) in that *Eoneureclipis* is a sister genus to *Lype*, and these two genera are the sister group to the remaining Psychomyiidae genera rather than the sister genus to all other Psychomyiidae and Xiphocentronidae genera as Silva suggested. However, discussing the relationship among psychomyiid genera based only on mt*COI* data is not reliable enough. In this study, the additional data of three species support the monophyly of each species. Unfortunately, we failed to obtain molecular data from *E. foraminulatus* sp. nov. and *E. spinosus* sp. nov.; therefore, we are unable to conduct a complete phylogenetic analysis on the relationships of the psychomyiid genera. More samples are needed to complete the analysis.

## Figures and Tables

**Table 1:** World list of *Eoneureclipsis* species.

Number	Species	Distribution
1	*E. jianfenglingensis* sp. nov.	China: Hainan
2	*E. foraminulatus* sp. nov.	China: Guangxi
3	*E. spinosus* sp. nov.	China: Guangxi, Guangdong
4	*E. gei* sp. nov.	China: Fujian
5	*E. malchidael* Malicky, 2020	China: Guangdong
6	*E. hainanensis* Mey, 2013	China: Hainan
7	*E. sarach* Malicky, 2014	China: Taiwan
8	*E. montanus* Torii & Nishimoto, 2011	Japan: Honshu
9	*E. okinawaensis* Torii & Nishimoto, 2011	Japan: Okinawa
10	*E. shikokuensis* Torii & Nishimoto, 2011	Japan: Shioku
11	*E. yaeyamaensis* Torii & Nishimoto, 2011	Japan: Yaeyama Islands
12	*E. alekto* Malicky & Chantaramongkol, 1997	Thailand
13	*E. chinachotiae* Malicky &Laudee, 2020	Thailand
14	*E. nykteus* Malicky & Nawvong, 2004	Thailand
15	*E. querquobad* Malicky & Chantaramongkol, 1989	Thailand
16	*E. afonini* Arefina-Armitage & Armitage, 2015	Vietnam
17	*E. quangi* Malicky, 1995	Vietnam
18	*E. tieni* Malicky, 1995	Vietnam
19	*E. akrichalakchmi* Schmid, 1972	India: Manipur
20	*E. varsikiyja* Schmid, 1972	India: Assam
21	*E. sebulon* Malicky, 2009	Laos
22	*E. pravrisija* Schmid, 1972	Myanmar
23	*E. limax* Kimmins, 1955	Malaysia: Sarawak

**Table 2:** Information on the collection of 8 specimens.

Species	Number of Specimens	Collection Site	Collection Date	Molecular Sample
*E. jianfenglingensis* sp. nov.	1 male	Jianfeng Town, Ledong County, Hainan	16 Apr 2019	yes
*E. jianfenglingensis* sp. nov.	1 male	Jianfeng Town, Ledong County, Hainan	27 Jul 2022	no
*E. foraminulatus* sp. nov.	1 male	Shangsi County, Fangchenggang City, Guangxi	06 Jun 2004	no
*E. spinosus* sp. nov.	2 males	Huanjiang County, Hechi City, Guangxi	15 Jun 2004	no
*E. spinosus* sp. nov.	1 male	Ruyuan County, Shaoguan City, Guangdong	21–22 May 2004	no
*E. gei* sp. nov.	1 male	Xingcun Town, Wuyishan City, Fujian	16 Jul 2022	yes
*E. hainanensis* Mey, 2013	1 male	Jianfeng Town, Ledong County, Hainan	28 Jul 2022	yes

**Table 3:** PCR primers used to sequence mt*COI* genes of *Eoneureclipsis* species in this study.

Primer	Sequence	Reference
LCO1490	GGTCAACAAATCATAAAGATATTGG	Folmer et al., 1994 [23]
HCO2198	TAAACTTCAGGGTGACAAAAAATCA	Folmer et al., 1994 [23]

## Data Availability

The voucher specimens from this research were deposited in the Insect Classification and Aquatic Insect Laboratory, College of Plant Protection, Nanjing Agricultural University, Nanjing, China.

## Supplementary Materials

The following Supplementary Materials can be downloaded at: https://www.mdpi.com/article/10.3390/insects14020158/s1. Table S1: Kimura 2-parameter pairwise genetic distances based on mt*COI* barcodes of Psychomyiidae.
